# Assessment of the preventive effects of Nd:YAG laser associated with fluoride on enamel caries using optical coherence tomography and FTIR spectroscopy

**DOI:** 10.1371/journal.pone.0254217

**Published:** 2021-07-07

**Authors:** Marcia Cristina Dias-Moraes, Pedro Arthur Augusto Castro, Daísa Lima Pereira, Patrícia Aparecida Ana, Anderson Zanardi Freitas, Denise Maria Zezell

**Affiliations:** 1 Center for Lasers and Applications, Instituto de Pesquisas Energeticas e Nucleares, IPEN—CNEN/SP, Sao Paulo, SP, Brazil; 2 Center for Engineering, Modelling and Applied Social Sciences, Universidade Federal do ABC, Sao Bernardo do Campo, SP, Brazil; University of Houston, UNITED STATES

## Abstract

**Objective:**

This *in vitro* study characterized and monitored, by Optical Coherence Tomography (OCT) and Fourier Transformed Infrared Spectroscopy (FTIR), the effects of the association of acidulated phosphate fluoride gel (APF-gel) and Nd:YAG (neodymiun:yttrium-aluminum-garnet) laser, as sequencial treatments, in the prevention of incipient enamel caries lesions.

**Methods:**

120 human enamel samples were randomized into 3 groups (n = 40): APF-gel (1.23% F^-^, 4 min.); Laser+APF (Nd:YAG laser irradiation—0.6W, 84.9J/cm^2^, 10Hz, followed by APF-gel); and APF+Laser (APF-gel followed by laser irradiation). The samples were subjected to a 15-day pH-cycling, evaluated by OCT (quantification of optical attenuation coefficient–OAC) and FTIR (analysis of carbonate and phosphate content) before treatments, after treatments, and on the 5^th^, 10^th^ and 15^th^ days of pH-cycling. The statistical analysis was performed (α = 5%).

**Results:**

The Optical Attenuation Coefficient (OAC) assessed by OCT increases with the progression of demineralization, and the Laser+APF presented the highest values of OAC in 10^th^ and 15^th^ days of pH-cycling. Nd:YAG decreased the carbonate content after treatment regardless of the application order of the APF-gel, while APF-gel did not interfere in the composition of enamel. The carbonate content was also changed in the first 5 days of the pH-cycling in all groups.

**Conclusion:**

Nd:YAG laser irradiation before or after the application of APF-gel did not influence the appearance of incipient caries lesions, showing no synergistic effect. Regardless of the application order of the APF-gel, laser irradiation reduces the carbonate content of the enamel, which also changes during the demineralization process. However, irradiation before the application of APF-gel increased the speed of progression of the lesions, which positively impacts public health as it can prevent caries disease, even in high risk individuals. OCT and FTIR are suitable for assessing this effect.

## Introduction

Dental caries is among the most prevalent multifactorial oral disease worldwide. Despite the availability of preventive therapies and the widespread use of fluoridated products, the world prevalence of untreated caries lesions, considering all ages, was estimated at 2.4 billion people, in 2017 [1, 2], which reflects the need for greater investment in strategies that minimize the incidence of lesions in certain groups of the population, especially people at high risk for caries who cannot go to the dentist very often.

Advances in restorative dentistry have highlighted the importance of reliable and minimally invasive methods to improve the remineralization processes, especially in high-risk individuals [3]. Among such strategies, the use of fluoride is still considered the most effective measure in the control of caries disease, taking into account the individual (dentifrices, mouthrinses, and topical application) or the public level (water supplies) [4]. The presence of fluoride in adequate concentration in the saliva or on the surface of the hydroxyapatite (HAP) crystals as calcium fluoride-like material (CaF_2_-like material), before or during a demineralization process, enhances remineralization and inhibits demineralization [5].

Studies involving high-power lasers have contributed to the development of clinical techniques to treat and prevent caries lesions [6–9]. To obtain an effective preventive action, it is necessary that the dental hard tissue (enamel or dentin) is chemically modified to be more resistant to demineralization. These modifications are dependent on the thermal effect promoted by high-power lasers, whose dosimetric parameters as wavelength, repetition rate, energy density, peak power and cooling system, are essential for such changes to be obtained in the absence of deleterious effects [10, 11]. Among the lasers used to prevent caries, the Nd:YAG 1064 nm has shown good results in *in vitro* [12, 13] and clinical studies [14]. The association with topical application of fluoride has been the best strategy, since laser irradiation can favor the formation of CaF_2_ and guarantee a more lasting effect [15].

On the other hand, a major concern in Cariology is the early diagnosis of incipient lesions, which allows preventive strategies to be carried out in a non-invasive manner, favoring the remineralization of the lesions. Based on the chemical and morphological changes induced by demineralization, the initial caries diagnosis involves the visual and tactile examinations of the lesion, an estimate of its depth, and degree of demineralization of these tissues accordingly to an experienced examiner [16]. Monitoring of a treatment is also important, which allows new interventions to be carried out [17]. In this way, the diagnosis and monitoring of lesions by optical techniques becomes quite advantageous, considering that these techniques are independent of the professional’s subjectivity, as well as allowing real-time assessment in a non-invasive way. Of the optical techniques commonly used, optical coherence tomography (OCT) stands out for its easy use and the possibility of high-resolution imaging of hard tissues [18, 19]. OCT is a high-resolution interferometric technique that provides quantitative outcomes and has the capability to produce cross-sectional images in real-time, without contact, allowing an analysis of the health of biological tissues, with the advantage of the use of non-ionizing radiation [20]. The current techniques of image processing, by the determination of the optical attenuation coefficient [21], already make this technique to be used clinically for evaluation and monitoring of carious lesions, as well as to detect the effectiveness of different preventive strategies, including the use of high-power lasers [12].

Another optical technique of interest is the Fourier transformed infrared (FTIR) spectroscopy, which may provide qualitative information about the chemical composition of tissues in a non-invasive way at a molecular level, allowing the analysis of structural and chemical changes caused by pathologies, like neoplasia and osteoporosis, aging, and radiation [22]. This optical technique was also successfully used to detect the changes promoted by laser irradiation in hard tissues and to monitor caries and erosion lesions [11, 14, 23, 24].

Although the Nd:YAG laser associated with acidulated phosphate fluoride (APF) can be effective in preventing incipient enamel caries lesions, there are no studies that relate optical to chemical changes promoted by different forms of association over a prolonged period of cariogenic challenge, what motivated us to carry out the present study.

## Material and methods

### Experimental design

**Fig 1:**
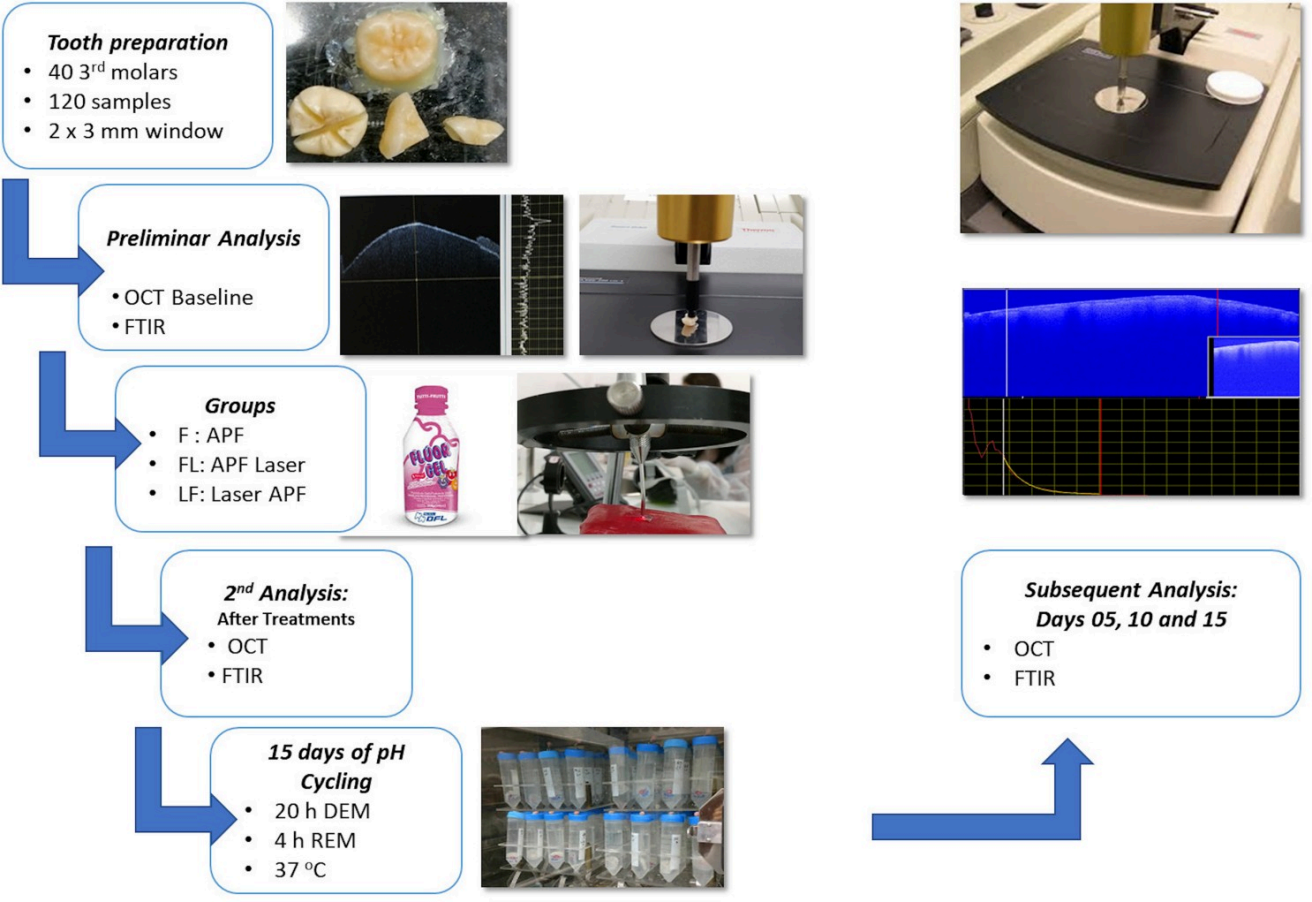
Study design.

The study protocol was approved by the Human Research Ethics Committee of the School of Dentistry of the University of Sao Paulo: number 529.666, 13/02/2014 National Code: CAAE 24791613.8.0000.5473. The specimens were obtained from the University biobank, and analysed anonymously. In a blind *in vitro* study, 120 human dental crown parts, obtained from 40 healthy non-erupted third molars, were examined on stereomicroscope to verify the absence of cracks and irregularities, and randomly distributed into three experimental groups, to compensate for their biological heterogeneity, with 40 enamel samples in each group as seem in Fig 1. The samples were embedded in acrylic resin and flattened to avoid changes in its position, protected with resistant acid varnish, except for a standardized exposed enamel window of 2 x 3 mm, not polished, to standardize the active area of the samples, where the treatments for the prevention of demineralization were performed: group APF—Application of APF-gel (acidulated phosphate fluoride gel, 1.23% fluoride, pH 3.6–3.9 Flu Gel—New DFL—Brazil); group Laser+APF—irradiation with Nd:YAG laser, followed by application of APF-gel, and group APF+Laser—APF-gel followed by Nd:YAG laser irradiation. APF-gel was applied over the samples with a cotton swab for 4 minutes, rinsed with Milli-Q water for 30 seconds and gently air dried [25]. The samples were washed with Milli-Q water and were kept in humid environment under refrigeration until the beginning of the experiments.

For irradiations, the Nd:YAG laser was used (Power Laser TM ST6, Lares Research, Chico, CA, USA); this equipment operates at wavelength of 1.064 μm, temporal width of 120 μs and the energy was delivered through a fiber optic system with 300 μm spot size. The irradiations were performed by hand, in contact mode, under the conditions of 0.6 W, 60 mJ per pulse, energy density of 84.9 J/cm^2^, and 10 Hz repetition rate. Before irradiation, the energy per pulse was calibrated by an energy/power meter (FieldMaster, Coherent, Santa Clara, CA, USA) and the surfaces of the samples were covered with a thin layer of a photoabsorber composed of triturated coal diluted in equal parts of deionized water and 99% ethanol [26]. After treatments, all samples were kept in humid environment under refrigeration until the next phase.

All samples were submitted to a 15-day pH-cycling model to simulate the process of demineralization and remineralization in the oral cavity to simulate caries lesions, according to Argenta et al. (2003) for 15 days [27]. The pH-cycling process was performed with the immersion of each sample individually in 38,22 ml of a demineralizing solution composed of 2.0 mM of calcium; 2.0 mM phosphate; 0.03 ppm fluoride in 0.075 M acetate buffer (pH 4.3) for 3 h. After this period, samples were washed with Milli-Q water for 15 s, dried with an absorbent paper and individually immersed in 19,08 ml of a remineralizing solution composed of 1.5 mM calcium, 0.9 mM phosphate, 150 mM potassium chloride, 0.05 ppm F- in 20 mM in cacodylate buffer (pH 7.4) for 20 h, and stored in an oven at 37°C to simulate the oral environment [27].

The optical and compositional effects promoted by all treatments, as well as the monitoring of demineralization process were evaluated by FTIR and OCT in five different moments: before treatments (baseline), after treatments, and in the 5^th^, 10^th^ and 15^th^ days of the pH-cycling. An individual statistical analysis for each variable response (composition and optical attenuation coefficient) was performed at the level of significance of 5%. For the statistical analysis, each treatment was considered a separate block, and the experimental unit was the sample (n = 40).

### FTIR analysis

The Fourier transform infrared spectroscopy in attenuated reflectance mode (ATR-FTIR, Thermo Nicolet 6700, Waltham, MA) was used for compositional analysis. The spectra of each sample were collected with 4.0 cm^−1^ resolution with 100 scans in the wavenumber range of 4000 to 600 cm^−1^. After the post spectroscopic offset-correction in 600–1200 cm^-1^, the ATR-FTIR spectra were smoothed by Savitzky-Golay algorithm with a polynomial of second order in 11 points window size. The ATR-FTIR spectral data with high signal-to-noise ratio were min-max normalized, where peak intensities should be scaled by setting the minimum absorbance unit to 0 and the maximum to 1 [28].

### OCT analysis

**Fig 2:**
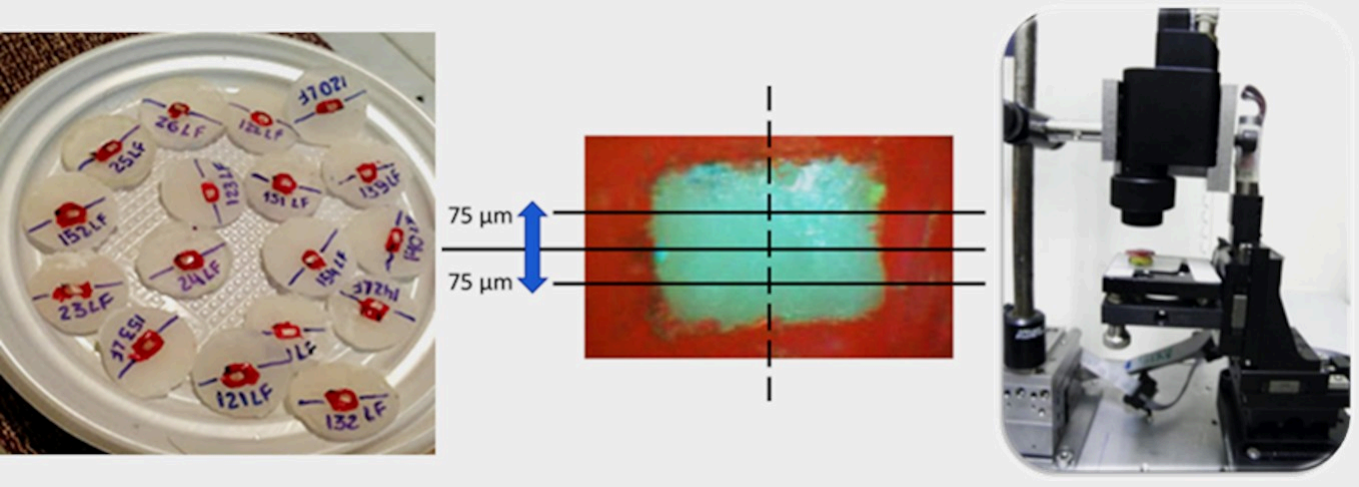
Positioning of samples for OCT measurements. A) Samples embedded in acrylic resin; B) Regions where measurements were performed: at the center of the ROI and 75 μm from the center in opposite directions; C) OCT probe fixed to prevent changes in measures through the experiment.

The optical analysis was performed using an OCT equipment (OCP930SR, Thorlabs Inc., Nelson, NJ), with the following characteristics: light source—SLED (super luminescent light-emitting diode), λ = 930 nm; 2 mW power; Lateral resolution: 6.0 μm; Depth resolution in air: 6.0 μm; Numbers of images per second: 3 fps (2000x512 pixels). A 2000 μm Region of Interest (ROI) was selected to be analyzed at the center of the samples at different times, using a home software developed in LabVIEW 8 (National Instruments) environment to obtain the total optical attenuation coefficient (OAC). Three images of each sample were collected at each time: in the central area of the sample, and 75 μm from the center in opposite directions. The OCT probe was fixed during the analyses. Also, a line was drawn and marks were made with a drill, laterally in the acrylic resin to allow a precise location of the center of the sample in the different times of the experiment (Fig 2).

**Fig 3:**
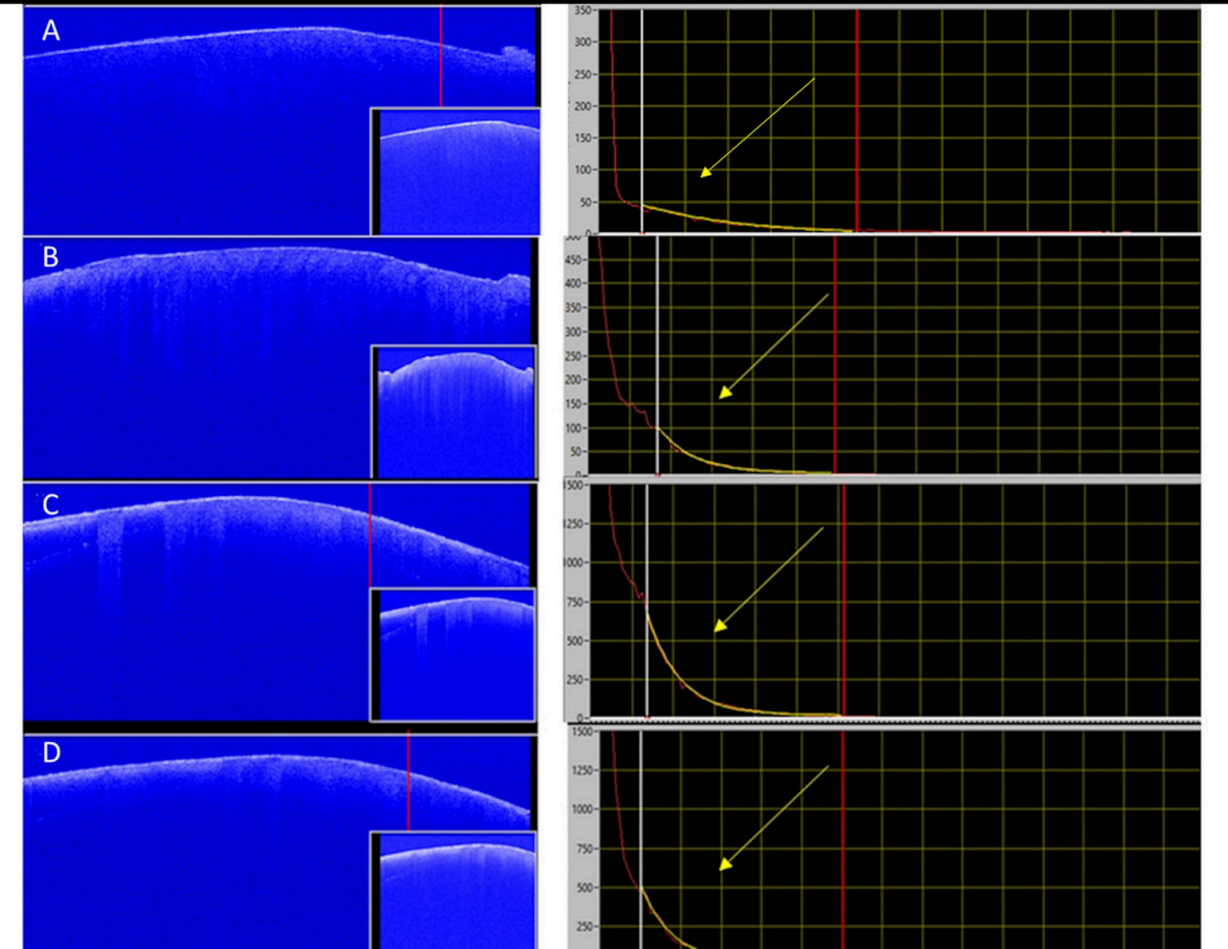
Exponential decay of the OCT signal obtained from one sample through the pH cycling (yellow arrows). A) Baseline B) Day 5; C) Day 10; D) Day 15. The software calculated the optical attenuation coefficient (OAC) of the ROI (Region of Interest—delimitated between the white and red lines in the left), as seen as a yellow curve in the right side.

The sweeping motion was perpendicular to the unprotected area in the samples, and an image of the cross-section of the geometric center was created. The images were used to assess the changes in the signal collected due to demineralization by the analysis of the optical attenuation coefficient (OAC) as stablished before [20], as seem in Fig 3. The considered value is an average of the OAC of 3 acquired B-Scans in the ROI: the first at the center of the sample, and two B-Scans 75 μm from the center, in opposite directions (as seem in Fig 2).

### Statistical analysis

The data of compositional analysis and the optical attenuation coefficient were statistically analyzed using the software GraphPad Prism for Mac (USA), with significance level set at p<0.05. Before the statistical analysis, the homogeneity and normality of the variances of experimental data were tested (Levene and Shapiro-Wilk).

## Results

**Fig 4:**
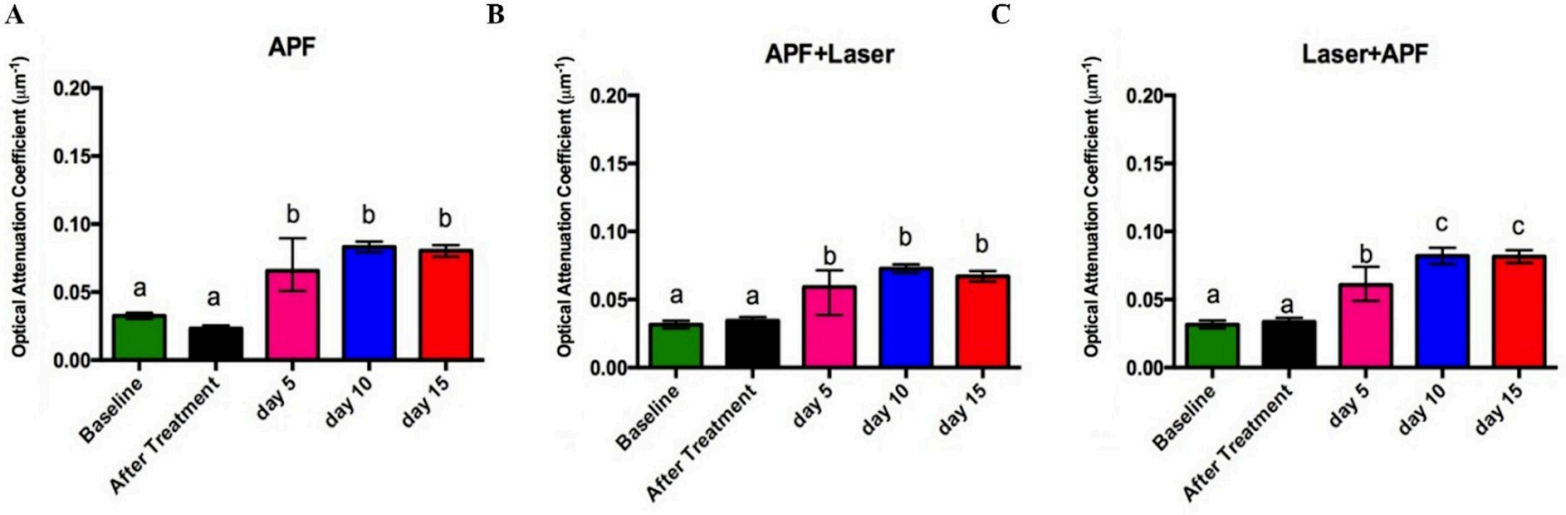
Average Optical Attenuattion Coefficient (OAC) obtained from the attenuation of the OCT signal of the samples over time. A) APF Group; B) APF+Nd:YAG Laser Group; C) Nd:YAG Laser+APF Group.

The Fig 4 shows the mean values (±SD) of OAC obtained for all experimental groups. It is possible to notice that none of the treatments changed the mean OAC values when compared with those observed for the untreated samples (baseline).

However, the pH-cycling significantly increased the OAC values in all experimental groups when compared to the baseline (Student paired t-Test, p < 0.001 in the comparisons for all experimental groups), and there were also no significant differences between the different pH-cycling periods in groups treated with APF or APF+Laser (Kruskal-Wallis + Student Newman-Keuls test, p > 0.05 for all comparisons). However, in the group treated with Laser+APF, there is a significant increase in mean OAC values after 10 and 15 days of pH-cycling when compared to the “baseline”, “after treatment” and “day 5 of pH-cycling” of this same group (Kruskal-Wallis + Student Newman-Keuls test, p < 0.02 for all comparisons); still, the average OAC values observed on days 10 and 15 of pH-cycling are not different in this experimental group (p = 0.67). When comparing the OAC values obtained after 10 and 15 days of pH-cycling among the three experimental groups (APF, APF+Laser and Laser+APF), however, statistically significant differences were observed only in the comparison after 15 days of pH-cycling, when the group treated with APF+laser showed lower mean value of OAC when compared with the other experimental groups (p = 0.0678 in day 10 and p = 0.04 in day 15 of pH-cycling, Kruskal-Wallis + Student Newman-Keuls test).

**Fig 5:**
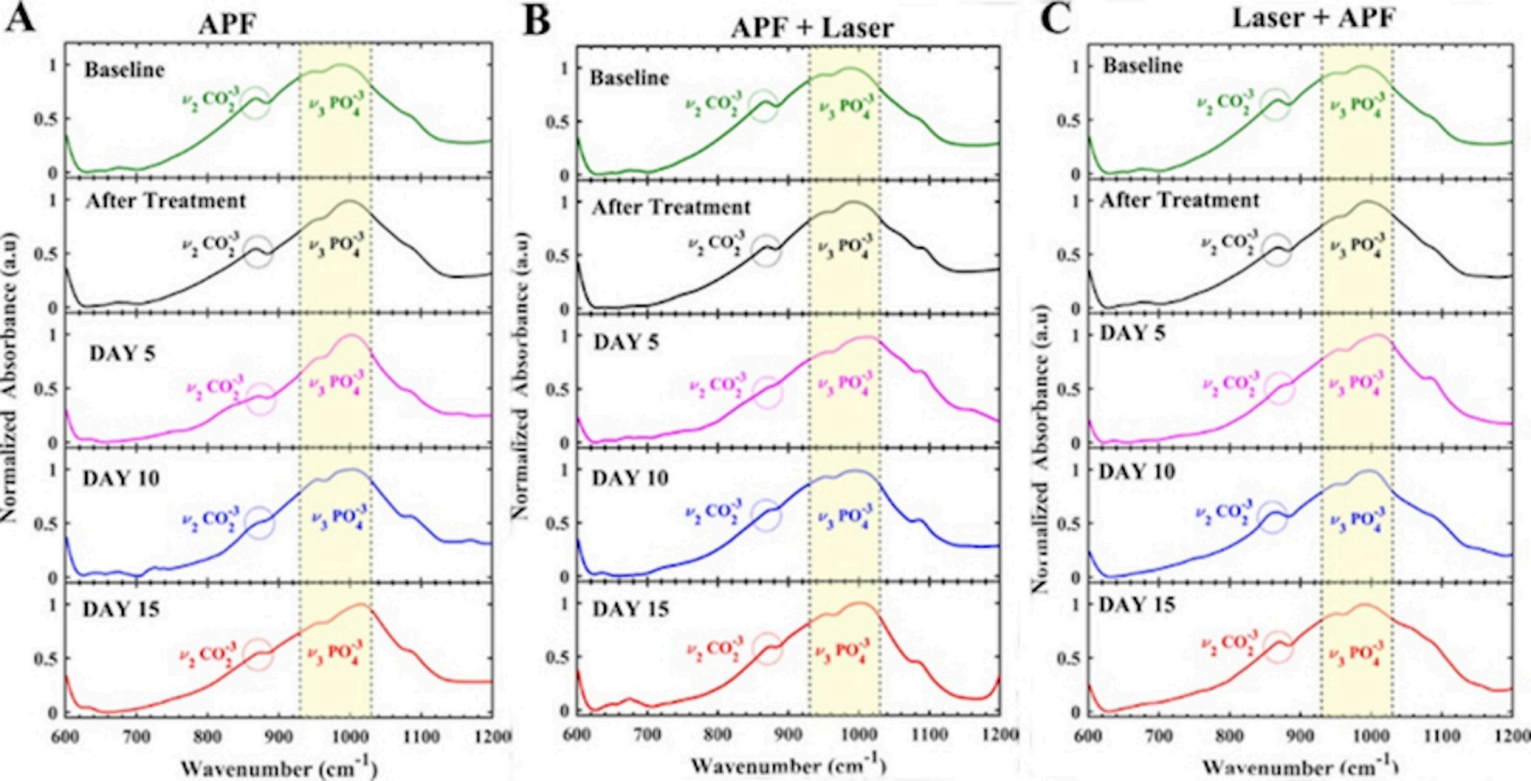
Representative spectra of each group over time, min-max normalized. A) APF Group; B) APF+Nd:YAG Laser; C) Nd:YAG Laser+APF.

**Fig 6:**
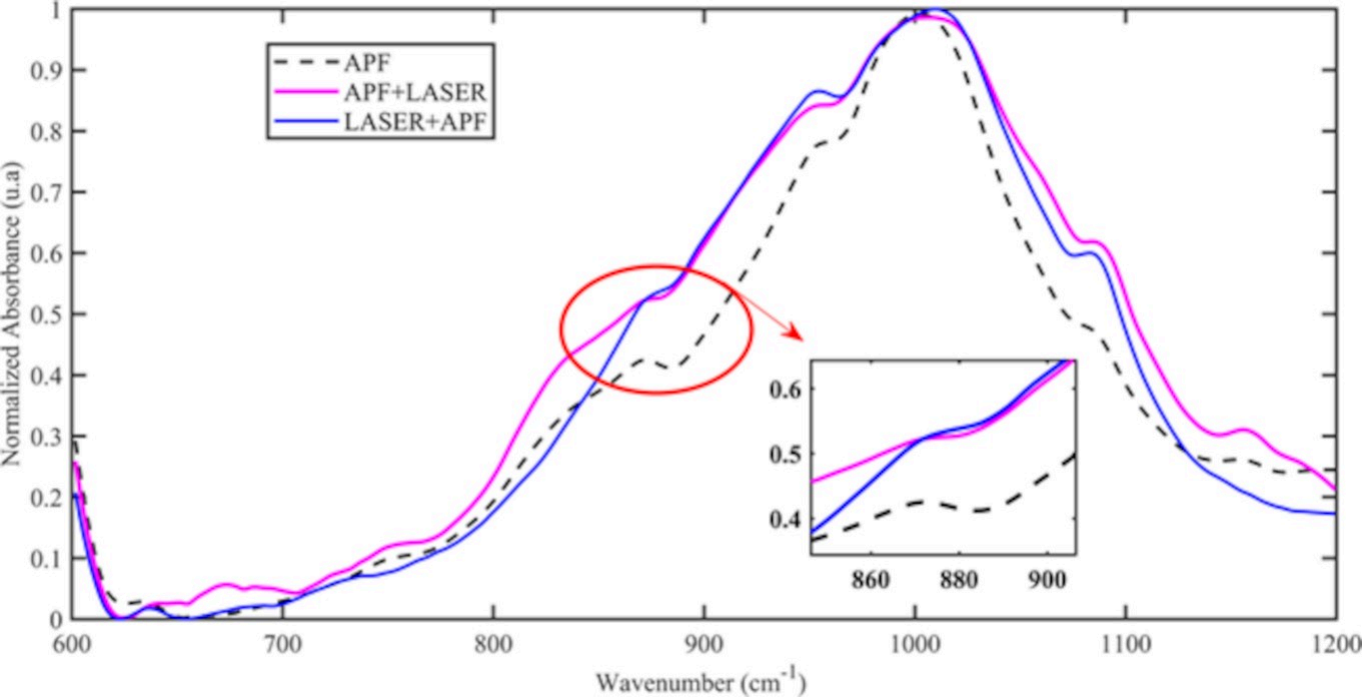
5th day–average spectra.

Concerning the compositional analysis, the representative spectra (Fig 5) of baseline (untreated enamel, day 0), after treatment and during pH-cycling (day 5, day 10 and day 15) show phosphate (ν_3_ PO_4_^-3^) and carbonate (ν_2_ CO_2_^-3^) bands. According to Fig 5, the spectra exhibited no changes associated to shift bands, or the appearance or disappearance of infrared bands. A decrease in the carbonate content was noticed in APF+Laser and Laser+APF groups immediately after treatments, but not after treatment with APF-gel. A carbonate band shape alteration was noticed in APF+Laser and Laser+APF groups, but not in the APF-gel group (Fig 6). On the 5^th^ day of pH-cycling, a decrease in the proportion between the carbonate content and the phosphate content was observed in all experimental groups (Fig 5); however, this proportion increases again on the 15^th^ day, mainly in the Laser + APF group.

## Discussion

The effects of the Nd:YAG laser for caries prevention are quite evident in the literature, with the durability of these effects being clinically evidenced for up to one year [13, 26]. To make the enamel more resistant to demineralization, the thermal effect promoted during irradiation modifies the enamel microstructure by evaporating water and carbonate, changing the organic matrix, forming new crystallographic phases such as tetracalcium phosphate, α- and β-tricalcium phosphate, bruxite, as well as increased hydroxyapatite crystals [15, 29, 30]. Nd:YAG laser can have a synergistic action with the topical application of fluoride, enhancing its preventive effects [26, 31–33]. Although there are other studies that do not show such a synergistic effect [34] such a strategy is particularly interesting in cases of patients at high risk for caries and can guarantee a more lasting preventive effect. However, the literature is conflicting regarding the order of treatments, that is, whether laser irradiation should be carried out before or after fluoride application [31, 35]. The present *in vitro* study sought to compare these strategies, evaluating the progression of incipient enamel caries lesions over a period of 15 days using a stablished pH-cycling model [21, 27, 36].

The use of non-invasive optical techniques proved to be interesting for such a comparison, as it made it possible to monitor the samples at several different times, without subjectivity and with greater accuracy [12, 21, 37]. The evaluation of the optical attenuation coefficient by OCT allows not only to diagnose the presence of the carious lesion but also to monitor its progression, thus evaluating the effectiveness of the treatments [38]. To this end, it is taken into account that the mineral loss process promotes increased enamel porosity, greater exposure of organic material and incorporation of water. Considering that OCT is an imaging technique that uses the backscattered signal, the increase in the intercrystalline spaces and the disorganization of the prismatic structure resulting from demineralization will result in a larger signal, which can be easily quantified through OAC [39]. Still, a previous study shows that OAC has a high correlation with the values of cross-sectional microhardness [21], a well-recognized technique to quantify mineral loss.

The present study showed that none of the treatments changed the OAC values, which indicates that they did not promote any optical changes in the tissue. This finding indicates that the treatments do not interfere in the analysis of OAC regarding the mineral content of the samples and in this way guarantees the reliability of future analyzes that have been carried out. Although irradiation with Nd:YAG laser, associated with the photoabsorber, promotes significant augment in surface temperature that can interfere with the amount of water in the enamel [15, 26, 40] the association of laser irradiation and posterior APF-gel application on the enamel surface did not allow such changes to occur significantly to the point of interfering with the tissue optics properties measured by OCT, in the present experimental conditions.

However, the pH-cycling promoted a significant increase in the average OAC values for all the groups evaluated, which implies demineralization in all samples [21]. In other words, none of the proposed treatments prevented the emergence of incipient carious lesions. This fact is expected, considering that, under acidic conditions, the apatite with or without fluoride will dissolve [41], and the formation of an incipient lesion will be inevitable. In this case, the presence of fluoride will promote the remineralization of the formed lesion and suppress further dissolution, that is, it will prevent the progression of the lesion [42]. In the present study, it was also possible to notice that there was no destruction of the enamel surface layer because this layer seems to be intact within the detection limit of the equipment (vertical resolution of 6 micrometers). Such findings corroborate the literature, emphasizing that the pH-cycling process promoted the removal of mineral and, with this, increased OAC [21].

Although the application of APF-gel results in large CaF_2_-like material formation, this material is weakly bound to the enamel surface [1]; thus, did not prevent the beginning of the demineralization process [43]. Still, after 5 days of pH-cycling, all treatments showed similar mean OAC values, which suggests that there was no synergistic action between laser and fluoride, under the conditions of the present study. This finding is in agreement with other studies [13, 15] and again it shows that, although a greater amount of CaF_2_-like material may have been formed, this amount has no additional effect in inhibiting demineralization. However, after 10 and 15 days of pH-cycling, the Laser+APF group showed the highest OAC values, which suggests greater progression of caries lesions in this group.

The literature shows different mechanisms of interaction between laser irradiation and APF-gel. Studies [32, 36, 44] show that morphological changes resulting from heating, such as roughness or microcracks, can favor the greater formation of CaF_2_-like material when irradiation is done before the application of APF-gel. Also, the adsorption of CaF_2_-like material on the enamel can be enhanced by the thermal action of the laser in the case of irradiation being carried out after APF-gel [45]. A literature study performed with CO_2_ laser also shows the potential for fluorapatite formation by laser heating [46], but this has never been evaluated with Nd:YAG laser. In the present study, if there was a greater formation of CaF_2_-like material in the Laser+APF group as it was determined before [15], this material may have been more dissolved in the pH-cycling solutions and removed when changing solutions, whereas, in the other experimental groups, this material may have become more retained on the surface and in this way the cariostatic effect was better evidenced.

The results observed in the compositional analysis corroborate the findings of OAC. In the present study, FTIR was used to assess the effects of demineralization on enamel; considering that the mineral loss implies a decrease in the content of carbonated hydroxyapatite, a reduction in the content of phosphate and carbonate (type A and type B, substituting for OH^-^ and phosphate ion, respectively) is expected in this process [47–49] and for this reason that only the region between 600 to 1200 cm^-1^ was considered. The topical application of APF-gel did not promote significant changes in the FTIR spectra of enamel, which agrees with previous findings [15] and indicates that APF-gel does not result in chemical changes or mineral loss of enamel. However, laser irradiation, before or after the application of APF-gel, promoted a reduction in the ν_2_ carbonate content, which indicates the thermal effect of the laser and corroborates previous results [12, 15, 30]. The literature state that the reduction of the carbonate content starts at a temperature above 100°C [29]; in this way, it can be inferred that such temperature was minimally reached during the treatments. Using the same laser parameters as those of the present study, Boari et al. (2009) reported temperature increases of up to 615°C on the enamel surface [26], which in fact corroborates the evidence of reduced carbonate content and increased resistance of the enamel irradiated to demineralization. The formation of new crystalline phases, changes in the size of the hydroxyapatite crystals, changes in water and organic content may have occurred, but they cannot be evidenced by the methodology carried out in this study.

FTIR analysis also detected a reduction in the proportion of ν_2_ carbonate in relation to the ν_3_ phosphate content after the beginning of pH-cycling (5^th^ day) in all experimental groups. Carbonate is a radical that can be incorporated into hydroxyapatite through substitutions of type A and type B by replacing the ions hydroxyl or phosphate during crystal development; the carbonate substitutions change the lattice parameter (reduces the crystallite size, the crystallinity and increase the crystal strain) and, in this way, this radical is the first to be lost during cariogenic challenge [50]. The carbonated hydroxyapatite is found in greater quantity on the surface of impacted teeth and it has greater solubility than pure hydroxyapatite in an acidic environment [47]. In this way, the reduction in the ν_2_ carbonate proportion related to the ν_3_ phosphate content in the first five days of the pH-cycling reflects the beginning of the demineralization process with the loss of carbonated hydroxyapatite. This fact corroborates the results of OAC and emphasizes that none of the treatments prevented the formation of incipient caries lesions even in the presence of fluoride, for the reasons discussed above.

At the end of the pH cycling, in the three experimental groups, it was possible to observe an augment in the proportion between ν_2_ carbonate and ν_3_ phosphate when compared with the proportion observed in the 5th day of pH-cycling. Literature studies indicate a significant increase in carbonate content going from outer to inner enamel [50, 51]; therefore, our FTIR results suggest the exposure of the internal enamel content due to the progression of caries lesions to deeper enamel in all groups. In this way, we can infer an enhance in mineral loss as the pH-cycling days increases. However, in Fig 5C, it is emphasized that this phenomenon starts on the tenth day of the pH-cycling in the group treated with Laser + APF, that is, the proportion between carbonate and phosphate is higher in this group when compared to the other experimental groups, in the same pH cycling period, which reinforces the OAC data and suggests that the speed of lesion progression was faster in Laser + APF experimental group. Therefore, again it is assumed that the CaF_2_-like material globules formed after treatment were more dissolved in the first days of the pH-cycling solutions, and for this reason the cariostatic effect was less in this group than the others.

Although an *in vitro* study allows precise control of all variables that may interfere with the analysis of results, especially those related to individuals (as in the case of *in situ* or clinical trials), the present study has some limitations. Despite of the fact that the manual laser irradiation mimic clinical situation, during research, in order to determine the best irradiation conditions and to standardize them, an automatic irradiation would be better. So, in this work the manual irradiation was a necessary limitation as the samples had the natural curvature of the teeth, not allowing using a motorized stage which would provide a flat irradiation patter with pulses side by side, leaving no space without laser pulse on it. As consequence, there were some non-irradiated areas on the enamel. Another limitation is related to the pH-cycling model, whose exchange or not of demineralizing and remineralizing solutions can alter the results, as well as the composition used may not reflect all the changes that occur in different individuals. It was decided to use a well-recognized pH-cycling model to minimize very incipient caries lesions and to determine the effects of low concentrations of fluoride, and the change in the total cycling time to 15 days was established in order to simulate a longer period of cariogenic challenge, therefore, related to individuals at high risk for caries. Although a synergistic effect of laser irradiation with APF-gel was not detected in this study, may be due to the limitations of the study, perhaps this effect can be detected in longer-lasting models, considering that laser irradiation acts on the composition of the substrate and not only in potentiating the effects of APF- gel. This study, however, sought to elucidate that the order of applications (Laser+APF or APF+Laser) may be a determining factor in the effectiveness of the treatment, and in fact it was observed that the greater formation of CaF_2_-like material may not result in better preventive effect. When used in conjunction with the topical application of APF-gel, the best performance of Nd:YAG laser may be to provide the best adsorption of CaF_2_-like material formed in the enamel, and thus favor the retention and performance of fluoride for a longer period of time. However, a long-lasting period of cariogenic challenge is necessary to verify whether there is a possibility of achieving a synergistic effect.

## Conclusion

With the limitations of this *in vitro* study, it was possible to conclude that none of the proposed treatments was able to prevent the enamel demineralization, and the Nd:YAG laser irradiation carried out before or after the application of APF-gel did not influence the appearance of incipient caries lesions, showing no synergistic effect. Regardless of the application order of the APF, laser irradiation reduces the carbonate content of the enamel, which also changes during the demineralization process. However, laser irradiation before the application of APF-gel increased the speed of progression of the caries lesions on enamel in 15 days of pH-cycling.

## Supporting information

S1 File (XLSX)

S2 File (XLSX)
